# The Changing Landscape of Systemic Treatment for Cervical Cancer: Rationale for Inhibition of the TGF-β and PD-L1 Pathways

**DOI:** 10.3389/fonc.2022.814169

**Published:** 2022-02-23

**Authors:** Michael J. Birrer, Keiichi Fujiwara, Ana Oaknin, Leslie Randall, Laureen S. Ojalvo, Christian Valencia, Isabelle Ray-Coquard

**Affiliations:** ^1^ Winthrop P. Rockefeller Cancer Institute, University of Arkansas Medical School, Little Rock, AR, United States; ^2^ Department of Gynecologic Oncology, Saitama Medical University International Medical Center, Saitama, Japan; ^3^ Gynaecological Cancer Program, Vall d’Hebron Institute of Oncology, Vall d’Hebron University Hospital, Barcelona, Spain; ^4^ Massey Cancer Center, Virginia Commonwealth University, Richmond, VA, United States; ^5^ EMD Serono Research & Development Institute, Inc., Billerica, MA, United States; ^6^ GINECO Group & Department of Medical Oncology, Centre Leon Berard, University Claude Bernard Lyon, Lyon, France

**Keywords:** cervical cancer, HPV, tumor microenvironment, TGF-β, PD-L1

## Abstract

Cervical cancer is one of the most common and lethal cancers among women worldwide. Treatment options are limited in patients with persistent, recurrent, or metastatic cervical cancer, with <20% of women living >5 years. Persistent human papillomavirus (HPV) infection has been implicated in almost all cases of cervical cancer. HPV infection not only causes normal cervical cells to transform into cancer cells, but also creates an immunosuppressive environment for cancer cells to evade the immune system. Recent clinical trials of drugs targeting the PD-(L)1 pathway have demonstrated improvement in overall survival in patients with cervical cancer, but only 20% to 30% of patients show overall survival benefit beyond 2 years, and resistance to these treatments remains common. Therefore, novel treatment strategies targeting HPV infection–associated factors are currently being evaluated in clinical trials. Bintrafusp alfa is a first-in-class bifunctional fusion protein composed of the extracellular domain of the TGF-βRII receptor (a TGF-β “trap”) fused to a human immunoglobulin G1 monoclonal antibody that blocks PD-L1. Early clinical trials of bintrafusp alfa have shown promising results in patients with advanced cervical cancer.

## Introduction

**Figure 1:**
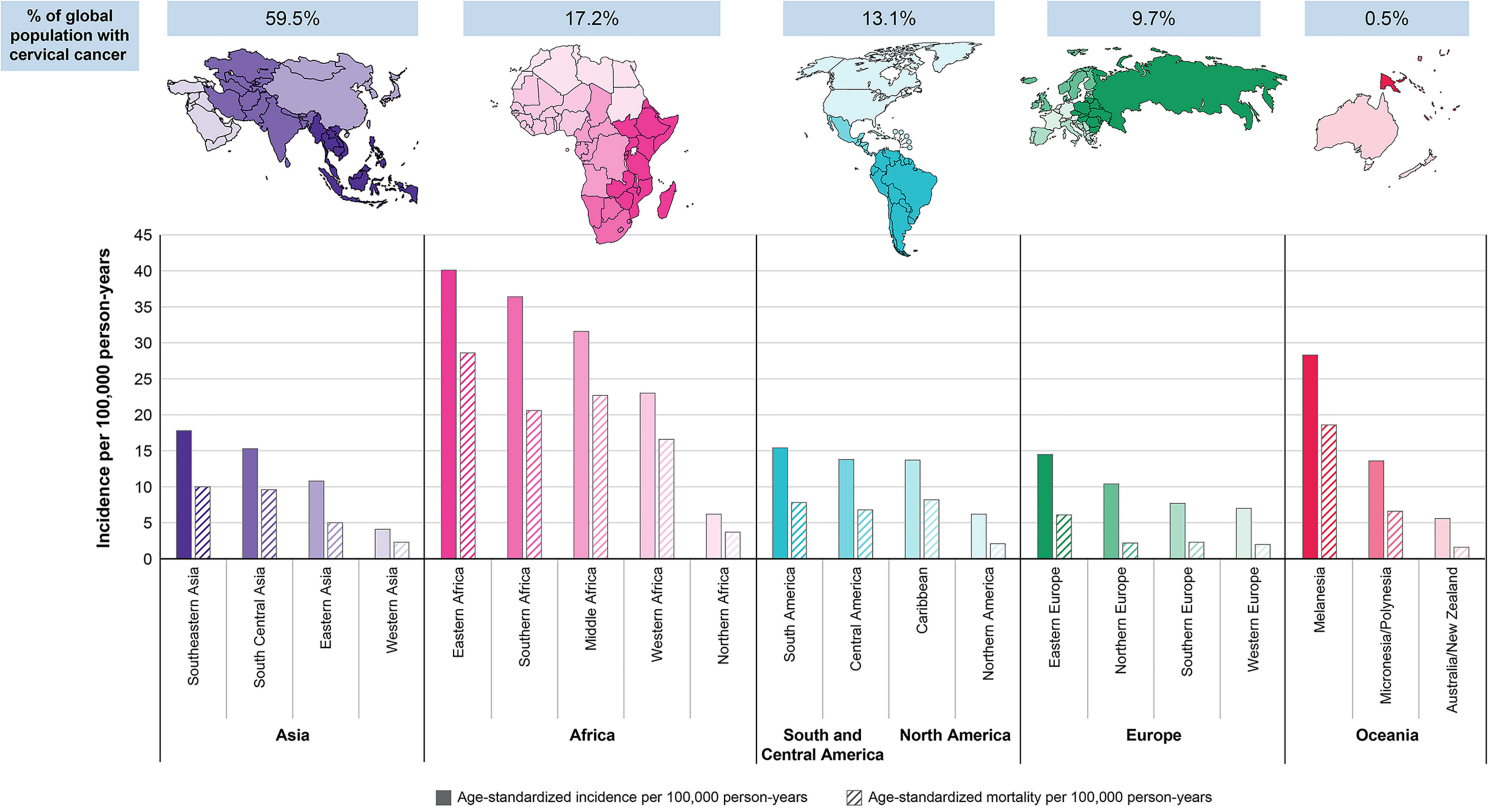
Region-specific age-standardized incidence and mortality rates for cervical cancer in 2020 (1).

With an estimated 604,000 new cases and 342,000 deaths globally in 2020, cervical cancer (malignant neoplasm of cervix uteri) is the fourth most commonly diagnosed cancer and the fourth leading cause of cancer-related death among women worldwide (1). In women younger than 45 years, cervical cancer was among the top 3 most common cancers in 79% of countries based on 2018 estimates (2) (**Figure 1**).

Cervical cancer is divided into 2 main histological subtypes: squamous cell carcinoma, which accounts for 70% to 80% of cervical cancers, and adenocarcinoma, which accounts for 20% to 25% (3, 4). Most data suggest that the prognosis of adenocarcinomas is worse than that of squamous cell carcinomas (3); however, these subtypes are treated similarly in the persistent, recurrent, or metastatic setting.

Cancer stage at diagnosis determines treatment options and influences the length of survival (5). Rates vary globally, with 12% to 36% of patients diagnosed with regional (stage II/III) disease and 5% to 16% diagnosed with distant or metastatic (stage IV) disease in the US, EU, and Japan (5–8). Among low-income and lower middle–income countries, cervical cancer is commonly diagnosed in the later stages of disease, most likely due to poor accessibility to preventive care (9, 10). Early cervical cancers and precancers usually do not cause noticeable signs or symptoms, which contributes to patients being diagnosed at a later stage (11). However, patients with recurrent or metastatic cervical cancer may have a variety of symptoms, including pain, anorexia, vaginal bleeding, cachexia, and psychological problems (12).

Women with unresectable, locally advanced cervical cancer are frequently treated using chemoradiotherapy (CRT); however, CRT is associated with late toxicities that impact quality of life, and recurrence after CRT carries a very poor prognosis (13–16). While advancements in CRT have improved overall survival (OS) in these patients, the risk of recurrence, typically within 2 years of diagnosis, is significant (3, 16, 17). In addition, treatment-related urinary, gastrointestinal, sexual, and neurological adverse effects can disrupt long-term quality of life (18). Survivors can also experience persistent emotional and psychological distress (19).

Persistent human papillomavirus (HPV) infection plays a crucial role in the pathogenesis of cervical cancers, particularly the oncogenic subtypes HPV16 and HPV18, which are responsible for 70% of cervical cancers (3, 10, 20–23). Cervical cancer cases occur at higher rates in countries that have not introduced an HPV vaccination program than in those that have implemented a vaccination program (10, 21). According to the World Health Organization’s Cervical Cancer Elimination Modelling Consortium, high HPV vaccination coverage has the potential to reduce cervical cancer mortality by almost 99% in most low-income and lower middle-income countries by the end of the century (9). Until then, understanding the biological role of HPV infection may be the key to identifying new treatments.

HPV infection mediates cervical cancer pathogenesis through various pathways. During persistent infection, viral DNA is randomly integrated into the host genome, leading to cellular immortalization and eventually to malignant progression (24). The combined action of HPV oncoproteins E6 and E7 can mediate the transformation of cervical cells into cancerous cells by driving cell proliferation, interfering with apoptosis, and promoting genomic instability; their expression is required to maintain a proliferative and malignant phenotype (24). Chronic immunosuppression carries a higher risk for malignant transformation with persistent HPV infection (25).

Here, we discuss the current treatment options and the future of systemic treatment for patients with persistent (defined as cervical cancer that remains present after treatment for locally advanced disease), recurrent (defined as cervical cancer that has progressed after treatment for locally advanced disease), or metastatic cervical cancer. We focus on the role of HPV infection, which has been implicated in 99% of all cases of cervical cancer, in the underlying disease biology of cervical cancer (3).

## Current Treatment for Persistent, Recurrent, or Metastatic Cervical Cancer

Chemotherapy doublets, primarily platinum based, were the first-line standard of care in the persistent, recurrent, or metastatic setting for many years (3). While chemotherapy doublets may still be used, adding bevacizumab, an anti–vascular endothelial growth factor (VEGF) monoclonal antibody, to cisplatin-paclitaxel is the preferred first-line regimen for patients with persistent, recurrent, or metastatic cervical cancer (3, 26, 27). The preference for this regimen was based on the results of the phase 3 GOG 240 randomized trial, which showed that the addition of bevacizumab to a chemotherapy doublet (cisplatin-paclitaxel or topotecan-paclitaxel) significantly improved median OS (16.8 vs 13.3 months; p=0.0068) and median progression-free survival (PFS; 8.2 vs 6.0 months; *P*=0.0002) compared with a chemotherapy doublet alone (3, 26, 28, 29). Although the OS difference between bevacizumab plus topotecan-paclitaxel and topotecan-paclitaxel was not statistically significant, this regimen is considered an alternative to cisplatin-paclitaxel plus bevacizumab in patients who are not eligible for cisplatin (27–30). The addition of bevacizumab to the chemotherapy doublet increased the incidence of grade ≥3 thromboembolic events, grade ≥2 hypertension, and fistulae occurrence, warranting more vigilant patient monitoring (3, 29). Carboplatin is routinely used instead of cisplatin in some countries and for specific patient subpopulations, such as those with renal impairment and those who have experienced relapse after prior cisplatin (31, 32). The use of carboplatin either as part of a doublet or with bevacizumab was evaluated in two trials [phase 3 JCOG0505 trial (31) and phase 2 CECILIA trial (32)]. Based on these data, carboplatin in combination with bevacizumab and paclitaxel is also considered an alternative regimen (26, 31, 32).

Despite the current best treatment of platinum-based doublet chemotherapy with bevacizumab, data from the GOG 240 trial showed a median PFS of 8.2 months in patients with persistent, recurrent, or metastatic cervical cancer, and >60% had disease progression within 12 months (29). In addition, median OS was 16.8 months, and the 5-year OS rates were <20%, irrespective of the chemotherapy used (29).

Recently, the US Food and Drug Administration (FDA) approved pembrolizumab in combination with chemotherapy, with or without bevacizumab, as first-line treatment for patients with persistent, recurrent, or metastatic cervical cancer whose tumors express programmed death ligand 1 (PD-L1; combined positive score [CPS] ≥1) (33). This approval was based on the phase 3 KEYNOTE-826 trial, in which median PFS with pembrolizumab vs placebo was 10.4 vs 8.2 months (hazard ratio, 0.62; 95% CI, 0.50-0.77; *P*<0.001) and median OS was not reached vs 16.3 months (hazard ratio, 0.64; 95% CI, 0.50-0.81; *P*=0.0001) (34, 35). Median duration of response (DOR) with pembrolizumab vs placebo was 18.0 vs 10.4 months and objective response rate (ORR) was 68.1% vs 50.2%.

No standard-of-care treatment for patients with recurrent or metastatic cervical cancer that progresses during or after platinum-containing chemotherapy is currently globally accepted (3, 26). Single-agent chemotherapy has limited efficacy in the second-line setting, with ORRs often ≤15%, median PFS <6 months, and median OS <10 months (3). Patients with recurrent or metastatic cervical cancer who experience disease progression on or after chemotherapy and whose tumors express PD-L1 (CPS ≥1) may receive pembrolizumab based on approval by the US FDA (33, 34, 36); however, there have been no approvals outside of the US to date. This accelerated approval was based on an ORR of 14.3% and a median DOR that was not reached (range, 4.1-18.6+ months) in the single-arm, phase 2 KEYNOTE-158 study (37). In an updated analysis with a median follow-up of 36.9 months, the ORR was 17.1% in the PD-L1–positive cohort with median DOR still not reached (38). Patients in the study with PD-L1–negative tumors did not respond to pembrolizumab. Immune-mediated adverse events (AEs), common with checkpoint inhibitor therapy, occurred in 25 patients (25.5%), with five patients (5.1%) experiencing one or more grade 3/4 events (37). No treatment-related deaths or immune-mediated AEs leading to death were reported. These data indicate the potential benefit of inhibiting PD-L1 but also the challenges in providing benefit to patients whose tumors do not express PD-L1. Promising results have also been reported recently with cemiplimab in second-line or later settings in patients with persistent, recurrent, or metastatic cervical cancer (39).

Given these data, while some advances in treatment have been made with anti-VEGF inhibitors and immune checkpoint inhibitors, much room exists for improving outcomes in patients with persistent, recurrent, or metastatic cervical cancer. Thus, a rational approach to identify new systemic therapies that address the underlying pathophysiology of the disease is needed.

## The Role of Transforming Growth Factor β (TGF-β) and PD-L1 in Cervical Cancer

HPV infections that persist and progress to cervical cancer evade the immune system, in part by creating an immunosuppressive environment (40–44). During HPV-associated cervical cancer progression, T helper 1 cytokines such as interferon-γ and interleukin (IL)-2 are downregulated, and T helper 2 cytokines such as TGF-β and IL-10 are upregulated; these cytokine changes produce a local immunosuppressive tumor microenvironment (TME) that inhibits antitumor immune responses (42–44). Genes responsible for promoting HPV gene expression, known as master regulators, are also associated with expression of molecules such as PD-L1, TGF-β, and IL-10, which also promote an immunosuppressive environment (45). Increases in programmed death 1 (PD-1) and PD-L1 (PD-[L]1) expression have been positively associated with HPV infection, increase in cervical intraepithelial neoplasia grade, and tumor metastasis in patients with cervical cancer (46).

**Figure 2:**
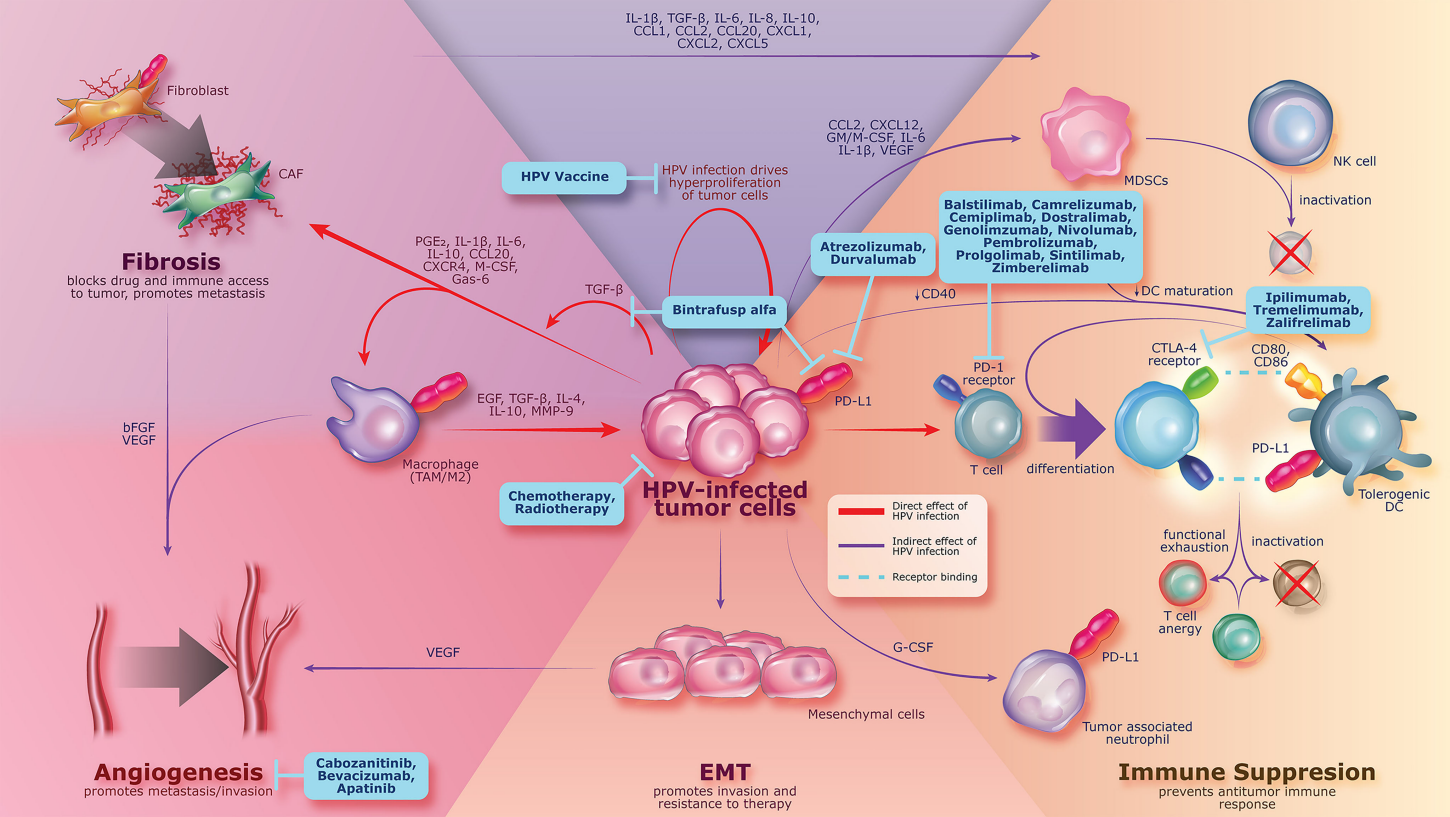
Persistent HPV infection leading to pathogenesis of cervical cancer by increasing the expression of immunosuppressive proteins (e.g., TGF-β, PD-1, and PD-L1), which can promote tumorigenesis, angiogenesis, fibrosis, and immune suppression. Current treatment strategies are shown in blue boxes and include agents with activity against cancer-associated changes related to HPV infection. bFGF, basic fibroblast growth factor; CAF, cancer-associated fibroblast; CCL, chemokine C-C motif ligand; CD, cluster of differentiation; CTLA-4, cytotoxic T-lymphocyte protein 4; CXCL, chemokine C-X-C motif ligand; DC, dendritic cell; EGF, epidermal growth factor; EMT, epithelial-mesenchymal transition; Gas, growth arrest-specific protein; G/M-CSF, granulocyte/macrophage colony-stimulating factor; HPV, human papillomavirus; IL, interleukin; MDSC, myeloid-derived suppressor cell; MMP, matrix metallopeptidase; NK, natural killer; PD-1, programmed death 1; PD-L1, programmed death ligand 1; PGE_2_, prostaglandin E_2_; TAM, tumor-associated macrophage; TGF-β, transforming growth factor β; VEGF, vascular endothelial growth factor.

Preclinical studies have shown that HPV oncoproteins E6 and E7 can upregulate TGF-β1 promoter activity (47) and can increase expression of immunosuppressive cytokines, resulting in overexpression of TGF-β, which stimulates survival and proliferation of cervical cancer cell lines (44, 48, 49). Aberrant TGF-β activation and signaling can promote tumorigenesis by stimulating epithelial-mesenchymal transition (EMT), angiogenesis, cancer-associated fibroblast activation, and immunosuppression within the TME (42, 50–52) (**Figure 2**). EMT has been implicated in cervical cancer and is shown to be associated with tumor progression and metastasis of primary tumors (53–56). TGF-β–mediated signaling in the TME upregulates the expression of VEGF, which promotes angiogenesis, facilitating nutrient exchange and metastasis that results in tumor progression (57, 58). TGF-β is a well-known regulator of fibrosis and can recruit cancer-associated fibroblasts to the TME and promote extracellular matrix remodeling, which has been implicated in increased cancer invasion, metastasis, and resistance to anticancer therapy in preclinical studies (59–67).

A positive correlation between HPV infection and TGF-β expression was observed in the serum and saliva of patients with oral squamous cell carcinoma, a cancer type also known to be associated with HPV infection (68). RNA sequencing analysis of HPV-positive oropharyngeal squamous cell carcinoma showed that high E6 expression was associated with poor survival (69). Hence, dysregulation of the TGF-β pathway due to HPV infection may play a critical role in carcinogenesis, not only in cervical cancer, but also in other HPV-associated cancers.

Increased TGF-β activity has also been implicated in resistance to immunotherapy in urothelial and colorectal tumors (70, 71). Elevated TGF-β levels in the TME represent a primary mechanism of immune evasion, which may play a role in mediating resistance to existing therapies targeting the PD-(L)1 pathway (42, 50, 72–77). Inhibition of TGF-β has been shown to enhance the response to PD-(L)1 blockade in preclinical studies by priming the TME and promoting T-cell infiltration (78). Furthermore, current standard-of-care therapies, including radiotherapy and chemotherapy, have been demonstrated to upregulate the TGF-β and PD-(L)1 pathways (79–82). In various mouse models of human cancer and in patient samples, radiation therapy and chemotherapy increase TGF-β signaling, expression, and protein secretion (79, 80, 83, 84). In addition, these treatments upregulate PD-1 expression on immune cells and PD-L1 expression on tumor cells, which could potentially mediate treatment resistance (81, 82, 85, 86). The important roles of TGF-β and PD-L1 in the development and progression of cervical cancer support the concept that these pathways are rational therapeutic targets.

## Future Treatment Options in Cervical Cancer Targeting HPV-Mediated Pathways

### First-Line Therapy

While many novel strategies that address the biology of cervical cancer are being explored with the aim of improving outcomes in the first-line setting (**Figure 2**), agents with activity against cancer-associated changes related to HPV infection are a key focus. These include anti–PD-(L)1 therapies, HPV therapeutic vaccines, and the bifunctional fusion protein bintrafusp alfa.

Based on the efficacy demonstrated with pembrolizumab and the significant role of PD-L1 in cervical cancer, various anti–PD-(L)1 agents are currently under investigation as first-line therapies in cervical cancer (**Tables 1** and **2**). Phase 3 trials of atezolizumab and prolgolimab (BCD-100) in combination with chemotherapy with or without bevacizumab and pembrolizumab with chemoradiotherapy are ongoing (**Table 2**).

**Table 1:** Efficacy and safety of immunotherapy agents as monotherapy or in combination with other therapies in cervical cancers.

Agents (MOA)	Study	Line of therapy	Phase	N	ORR, %	Median DOR, months	Median OS, months	TRAEs	
								Overall incidence and most common*, %	Grade 3/4 and most common*, %
**First-line therapies**									
Bintrafusp alfa (TGF-β “trap” + anti–PD-L1) + CT ± bevacizumab (87)	INTR@PID CERVICAL 046 (NCT04551950)	1L	1	17	Cohort 1A: 12.5 Cohort 1B: 11.1	–	–	Cohort 1A: 62.5; Stomatitis (37.5), anemia (25.0), diarrhea (25.0), dysgeusia (25.0), lipase increased (25.0); Cohort 1B: 100; Diarrhea (55.6), amylase increased (33.3), gingival bleeding (33.3), stomatitis (33.3)	Cohort 1A: 37.5; anemia (25.0), lipase increased (12.5), neutrophil count decreased (12.5), maculopapular rash (12.5); Cohort 1B: 22.2; anemia (11.1), rectal hemorrhage (11.1), vaginal bleeding (11.1)
ISA101 + CT (HPV vaccine) (88)	NCT02128126	1L	1/2	72	43	–	–	TEAEs: 87.5	TEAEs: 27.8
ISA101 + nivolumab (HPV vaccine + anti–PD-1) (89)	NCT02426892	1L/2L	2	1^†^	0	–	–	–	–
Nivolumab (anti–PD-1) (90)	CheckMate 358 (NCT02488759)	1L	1/2	4	25.0	Not reached	21.9^‡^	63.2^‡^ Diarrhea (21.1), fatigue (15.8), pneumonitis (10.5), abdominal pain (10.5), stomatitis (10.5), dry eye (10.5), arthralgia (10.5)	21.1* Diarrhea (5.3), pneumonitis (5.3)
Nivolumab 3 mg/kg + ipilimumab 1 mg/kg (anti–PD-1 + anti–CTLA-4) (91)	CheckMate 358 (NCT02488759)	1L	2	19	31.6	Not reached	Not reached	80.0	28.9
Nivolumab 1 mg/kg + ipilimumab 3 mg/kg (anti–PD-1 + anti–CTLA-4) (91)	CheckMate 358 (NCT02488759)	1L	2	24	45.8	–	–	82.6	37.0
Pembrolizumab (anti–PD-1) + CT ± bevacizumab (35)	KEYNOTE-826 (NCT03635567)	1L	3	308	65.9	18.0	24.4	97.1 Alopecia (55.7), anemia (48.5), nausea (33.9), diarrhea (24.8), peripheral neuropathy (24.4)	68.4 Anemia (24.8), neutrophil count decreased (13.0), neutropenia (12.1), platelet count decreased (6.8), thrombocytopenia (6.8), WBC count decreased (6.8)
**Second- or later-line therapies**									
**Monotherapies**									
Balstilimab (anti–PD-1) (92)	RaPiDS (NCT03894215)	2L	2	44	11.4	–	–	9.1	–
Balstilimab (anti–PD-1) (93)	NCT03104699	2L+	2	161	15.0	15.4	–	71.4 Asthenia (23.0), diarrhea (12.4), pruritus (11.8), fatigue (10.6)	11.8 Immune-mediated enterocolitis (3.1), diarrhea (1.9), hypokalemia (1.2), anemia (1.2)
Bintrafusp alfa (TGF-β “trap” + anti–PD-L1) (94)	INTR@PID 001 (NCT02517398)	2L+	1	25	28.2	11.7	13.4	84.6Dermatitis acneiform (20.5), anemia (17.9), epistaxis (17.9), maculopapular rash (15.4), pruritus (15.4)	20.5Anemia (2.6), colitis (2.6), gastroparesis (2.6), upper GI hemorrhage (2.6), keratoacanthoma(2.6), cystitis noninfective (2.6), hematuria (2.6), pneumonitis (2.6), rash macular (2.6)
Bintrafusp alfa (TGF-β “trap” + anti–PD-L1) (94)	NCI 012 (NCT03427411)	2L+	2	14					
BVAC-C (HPV vaccine) (95)	NCT02866006	2L	2a	21	21	18	–	–	–
Cemiplimab (anti–PD-1) (39, 96)	EMPOWER (NCT03257267)	2L+	3	304	16.4	16.4	12.0	56.7 TEAEs: anemia (25.0), nausea (18.3), fatigue (16.7), vomiting (16.0), decreased appetite (15.0), constipation (15.0)	14.7 TEAEs: anemia (12.0), asthenia (2.3), fatigue (1.3), neutropenia (1.0), vomiting (0.7)
LN-145 (autologous TILs) (97)	C-145-04 (NCT03108495)	2L+	2	27	44.4	Not reached	–	TEAEs: 100 Chills (77.8), anemia (55.6), diarrhea (51.9), pyrexia (51.9), thrombocytopenia (51.9)	TEAEs: 96.3 Anemia (55.6), thrombocytopenia (44.4), neutropenia (29.6), febrile neutropenia (29.6), leukopenia (22.2)
Nivolumab (anti–PD-1) (90)	CheckMate 358 (NCT02488759)	2L+	1/2	15	26.7	Not reached	21.9^‡^	63.2* Diarrhea (21.1), fatigue (15.8), pneumonitis (10.5), abdominal pain (10.5), stomatitis (10.5), dry eye (10.5), arthralgia (10.5)	21.1* Diarrhea (5.3), pneumonitis (5.3)
Nivolumab (anti–PD-1) (98)	NRG-GY002 (NCT02257528)	2L	2	25	4	3.8	14.5	84	Grade 3: 24 Grade 4: 8
Pembrolizumab (anti–PD-1) (99)	KEYNOTE-028 (NCT02054806)	2L+	1	24	17	5.4	11	75 Rash (21), pyrexia (17), fatigue (8), asthenia (8), constipation (8), diarrhea (8), dry mouth (8), anemia (8), proteinuria (8), dry skin (8), pruritus (8)	Grade 3: 21 Rash (8), proteinuria (4)
Pembrolizumab (anti–PD-1) (38)	KEYNOTE-158 (NCT02628067)	2L+	2	98	14.3	Not reached	9.3	65.3 Fatigue (11.2), hypothyroidism (11.2), decreased appetite (9.2), asthenia (8.2), diarrhea (8.2), hyperthyroidism (7.1)	12.2 Hepatitis, asthenia, diarrhea, pyrexia, arthralgia
Zimberelimab (anti–PD-1) (100)	NCT03972722	2L+	2	45	26.8	Not reached	–	80.0 Anemia (26.7), increased GGT (15.6), increased blood AP (13.3), proteinuria (13.3), UTI (11.1), asthenia (11.1)	37.8 Anemia (15.6), hypokalemia (4.4), increased GGT (2.2), UTI (2.2), asthenia (2.2)
**Combination therapies**									
Atezolizumab + bevacizumab (anti–PD-L1 + VEGFi) (101)	NCT02921269	2L+	2	11	0	–	8.9	Fatigue (36), increased AST (27), nausea (27), fever (27), increased ALT (18), diarrhea (18), dyspnea (18)^§^	36.4 Muscle weakness (9), peripheral sensory neuropathy (9), arachnoiditis (9), sensorineural hearing loss (9)
Balstilimab + zalifrelimab (anti–PD-1 + anti–CTLA-4) (92)	RaPiDS (NCT03894215)	2L	2	34	20.6	–	–	14.6	–
Balstilimab + zalifrelimab (anti–PD-1 + anti–CTLA-4) (102)	NCT03495882	2L+	2	155	25.6	Not reached	12.8	71.0 hypothyroidism (16.8), diarrhea (14.2), fatigue (11.6), nausea (9.0), hyperthyroidism (8.4), increased AST (8.4), pyrexia (8.4)	20.0 Increased ALT (2.6), diarrhea (1.9), anemia (1.3), platelet count decreased (1.3), immune-mediated enterocolitis (1.3), maculopapular rash (1.3), increased GGT (1.3)
Camrelizumab + apatinib (anti–PD-1 + VEGFR-2i) (103)	CLAP (NCT03816553)	2L+	2	45	55.6	Not reached	Not reached	95.6 Hypertension (84.4), anemia (60.0), proteinuria (55.6), increased AST (51.1), increased ALT (40.0)	71.1 Hypertension (24.4), anemia (20.0), increased GGT (15.6), fatigue (15.6), neutropenia (6.7)
Durvalumab + tremelimumab (anti–PD-L1 + anti–CTLA-4) (104)	NCT01975831	2L	1	13	0	–	–	–	–
Nivolumab 3 mg/kg + ipilimumab 1 mg/kg (anti–PD-1 + anti–CTLA-4) (91)	CheckMate 358 (NCT02488759)	2L+	2	26	23.1	14.6	10.3	80.0	28.9
Nivolumab 1 mg/kg + ipilimumab 3 mg/kg (anti–PD-1 + anti–CTLA-4) (91)	CheckMate 358 (NCT02488759)	2L+	2	22	36.4	9.5	25.4	82.6	37.0
Pembrolizumab + GX-188E (anti–PD-1 + HPV vaccine) (105)	KEYNOTE-567 (NCT03444376)	2L	2	52	31.1	–	16.7	32.7	3.8 Increased ALT (1.9), increased AST (1.9)
Simlukafusp alfa + atezolizumab (FAP–IL-2v + anti–PD-L1) (106)	NCT03386721	2L+	2	47	27	13.3	–	–	Grade 3: 63.8 Grade 4: 29.8
Sintilimab + anlotinib (anti–PD-1 + TKI) (107)	ChiCTR1900023015	2L+	2	42	56.4	–	–	Hypothyroidism (33.3), hypertension (23.8), AST (21.4), diarrhea (19.0), ALT (16.7)	Hypertension (4.8), hyponatremia (4.8), immune pneumonia (2.4), immune myocarditis (2.4)

*Most common preferred term refers to the five most common TRAEs, if reported.

^†^Part of a phase 2 trial enrolling 24 patients with HPV-positive cancers (most of which were SCCHN).

^‡^Includes all patients comprising those who received 1L and 2L+ treatment.

^§^TRAEs attributed to atezolizumab.

1L, first line; 2L, second line; ALT, alanine aminotransferase; AP, alkaline phosphatase; AST, aspartate aminotransferase; CT, chemotherapy; CTLA-4, cytotoxic T-lymphocyte protein 4; DOR, duration of response; FAP, fibroblast activation protein-α; GGT, γ-glutamyl transferase; GI, gastrointestinal; HPV, human papillomavirus; IL-2v, interleukin-2 variant; MOA, mechanism of action; NA, not applicable; ORR, objective response rate; OS, overall survival; PD-1, programmed death 1; PD-L1, programmed death ligand 1; SCCHN, squamous cell carcinoma of the head and neck; TEAE, treatment-emergent adverse event; TGF-β, transforming growth factor β; TIL, tumor-infiltrating lymphocyte; TKI, tyrosine kinase inhibitor; TRAE, treatment-related adverse event; UTI, urinary tract infection; VEGFi, vascular endothelial growth factor inhibitor; WBC, white blood cell.

**Table 2:** Ongoing clinical trials of immunotherapy agents in cervical cancers.

Agents (MOA)	Key inclusion criteria	Combination partner	Study	Phase	N	Locations	Primary completion date
**First-line therapies**							
Atezolizumab (anti–PD-L1) (108)	Female; age ≥18 y; persistent, recurrent, or metastatic SCC, AC, or AS; no prior systemic treatment	CT+ bevacizumab	BEATcc (NCT03556839)	3	404	US, Asia, and Europe	3/2023
Pembrolizumab (anti–PD-1)	Female; age ≥18 y; persistent, recurrent, or metastatic SCC, AC, or AS; no prior systemic treatment	CT + bevacizumab	NCT03367871	2	40	US	10/2022
Pembrolizumab (anti–PD-1)	Female; age ≥18 y; high-risk locally advanced cancer; SCC, AC, or AS; no prior systemic treatment	CRT	ENGOT-cx11/GOG 3047/KEYNOTE-A18 (NCT04221945)	3	980	Global	2/2024
Prolgolimab (BCD-100; anti–PD-1)	Female; age ≥18 y; recurrent, or metastatic cervical cancer; no prior systemic treatment	CT ± bevacizumab	FERMATA (NCT03912415)	3	316	Global	12/2024
Prolgolimab (BCD-100; anti–PD-1)	Female; age ≥18 y; persistent, recurrent, or metastatic SCC, AC, or AS; no prior systemic treatment	CT + bevacizumab	CAESURA (NCT03912402)	2	49	Russia	7/2020
Z-100 (Immune-modulator)	Female; age ≥21 y; FIGO stage IIIB; confirmed SCC; no prior systemic treatment	RT	NCT03476018	3	72	Vietnam	10/2021
**Second- or later-line therapies**							
AK104 (anti–PD-1 + anti–CTLA-4)	Female; age ≥18 y; recurrent or metastatic SCC or AC; ≥1 prior systemic therapy	None	NCT04380805	2	40	US and Australia	8/2021
Atezolizumab + KY-1044 (anti–PD-L1 + ICOSi)	Patients; age ≥18 y; metastatic cancers; anti–PD-(L)1 therapy naive and pretreated	None	NCT03829501	1/2	412*^,†^	Global	5/2023
Atezolizumab + MG1-E6E7 + Ad-E6E7 (anti–PD-L1 + anti–HPV oncolytic virus + HPV vaccine)	Age ≥18 y; recurrent or metastatic HPV-associated cancers; received prior therapy	None	Kingfisher (NCT03618953)	1	75^†^	US and Canada	4/2021
Atezolizumab + tiragolumab (anti–PD-L1 + anti-TIGIT)	Female; age ≥18 y; recurrent or persistent SCC or AC; ≥1 prior systemic CT	None	SKYSCRAPER-04 (NCT04300647)	2	160^‡^	Global	7/2023
Atezolizumab ± Vigil (anti–PD-L1 + TGF-β/GM-CSF vaccine)	Female; age ≥18 y; confirmed stage IIIB, IIIC, or IV or metastatic cancers; recurrent ovarian disease	None	NCT03073525	2	25^†^	US	1/2021
Bintrafusp alfa (TGF-β “trap” + anti–PD-L1)	Female; age ≥18 y; advanced, unresectable, or metastatic SCC, AC, or AS; progression after platinum therapy	None	INTR@PID CERVICAL 017 (NCT04246489)	2	146	Global	12/2021
Bintrafusp alfa (TGF-β “trap” + anti–PD-L1) (109)	Female; age ≥18 y; locally advanced or metastatic HPV-associated cancers; ≥1 prior anticancer therapy	None	NCI 012 (NCT03427411)	2	57^†^	US	12/2022
Dostarlimab + niraparib (anti–PD-1 + PARPi)	Female; age ≥18 y; recurrent or progressive cervical cancer; ≥1 prior systemic therapy	None	STAR (NCT04068753)	2	66	US	7/2023
Durvalumab + MEDI-0457 (anti–PD-L1 + HPV vaccine)	Age ≥18 y; recurrent or metastatic HPV-associated cancers; refractory or relapsed after standard therapy	None	NCT03439085	2	77^†^	US	12/2021
Durvalumab + tremelimumab (anti–PD-L1 + anti–CTLA-4)	Female; age ≥18 y; recurrent or metastatic gynecologic cancer; progression on platinum-based CT	RT	NCT03277482	1	32^†^	US	1/2022
Genolimzumab (GB-226; anti–PD-1)	Female; age ≥18 y; recurrent, or metastatic cervical cancer; progression after platinum therapy	None	NCT03808857	2	80^‡^	China	12/2020
Gemogenovatucel-T + durvalumab (TGF-β/GM-CSF vaccine + anti–PD-L1)	Female; age ≥18 y; locally advanced or metastatic cancers; treatment naive or resistant to anti–PD-(L)1 therapy	None	NCT02725489	2	13*^,†^	US	12/2019
NP137 + pembrolizumab (anti–Netrin-1 + anti–PD-1)	Female; age ≥18 y; recurrent cervical cancer; ≥1 prior CT regimen	CT	GYNET (NCT04652076)	1/2	240	France	10/2021
Pembrolizumab + cabozantinib (anti–PD-1 + VEGFRi)	Female; age ≥18 y; recurrent or persistent SCC, AC, or AS; prior systemic CT	None	NCT04230954	2	39^‡^	US	6/2021
Pembrolizumab + olaparib (anti–PD-1 + PARPi) (110)	Female; age ≥18 y; recurrent cervical cancer; ≥1 prior CT regimen	None	NCT04483544	2	48	US	11/2030
Tislelizumab + ociperlimab (anti–PD-1 + anti-TIGIT) (111)	Female; age ≥18 y; SCC, AC, or AS; ≥1 prior CT	None	AdvanTIG-202 (NCT04693234)	2	167	China, South Korea, and Taiwan	3/2022
Tucatinib + trastuzumab (tyrosine kinase inhibitor + HER2 inhibitor)	Female; age ≥18 y; metastatic cancer; ≥1 prior systemic therapy	None	SGNTUC-019 (NCT04579380)	2	270^†^	US	1/2023

*Some patients enrolled in this study will have received one or two prior lines of therapy for recurrent/metastatic cervical cancer.

^†^Not all patients enrolled in this study will have cervical cancer.

^‡^Patients must have PD-L1–positive tumors.

AC, adenocarcinoma; AS, adenosquamous; CRT, chemoradiotherapy; CT, chemotherapy; CTLA-4, cytotoxic T-lymphocyte protein 4; FIGO, Federation Internationale de Gynecolgie et d’Obstetrique; GM-CSF, granulocyte-macrophage colony-stimulating factor; HER2, human epidermal growth factor receptor 2; HPV, human papilloma virus; ICOSi, inducible T-cell costimulator inhibitor; MOA, mechanism of action; PARPi, poly (adenosine diphosphate–ribose) polymerase inhibitor; PD-1, programmed death 1; PD-L1, programmed death ligand 1; RT, radiotherapy; SCC, squamous cell carcinoma; TGF-β, transforming growth factor β; TIGIT, T-cell immunoreceptor with immunoglobulin and immunoreceptor tyrosine-based inhibition motif domains; VEGFRi, vascular endothelial growth factor receptor inhibitor.

In addition, several trials are examining novel combinations. One such novel combination includes the HPV vaccine ISA101, which initiates an immune response to HPV16 proteins E6 and E7 and presumably results in cells infected with HPV16 being recognized by the immune system (84). ISA101 in combination with chemotherapy has shown promising results in patients with advanced cervical cancer (88, 112). This vaccine is injected after initiation of chemotherapy at a time point when reduction in myeloid cells leads to decreased immunosuppression and a strong T-cell response to the ISA101 vaccine. Median OS was 16.8 months in 32 patients with a high HPV16-specific vaccine-induced immune response compared with 11.2 months in 32 patients with a low HPV16-specific vaccine-induced immune response in an open-label phase 1/2 study in patients with HPV-positive cervical cancer (88). Additionally, 31 of 72 patients (43%) had tumor regression; however, given that this was a single-arm study, it was not possible to assess whether antitumor activity was due to the impact of ISA101 plus chemotherapy or chemotherapy alone.

### Post Platinum-Based Therapy

The potential of anti–PD-(L)1 therapies in second and later lines of therapy for cervical cancer was demonstrated with the approval of pembrolizumab for patients with recurrent or metastatic cervical cancer who experience disease progression on or after chemotherapy and whose tumors express PD-L1. However, an unmet need remains because response rates with pembrolizumab were low and treatment eligibility is restricted based on PD-L1 expression. Several other anti–PD-(L)1 treatments and novel agents are being explored in this setting.

#### Monotherapies

Anti–PD-(L)1 therapies such as atezolizumab, durvalumab, nivolumab, and pembrolizumab have demonstrated strong efficacy in other tumor types but have limited efficacy as second-line treatment in cervical cancer, although safety profiles are consistent across tumor types. In small phase 1/2 trials in patients with cervical cancer, balstilimab, cemiplimab, nivolumab, pembrolizumab, and zimberelimab monotherapies have reported ORRs from 4% to 26.8%, and while median DORs were not reached in many trials, the median OS was approximately 1 year with most agents (**Table 1**).

Recently reported data from the phase 3 EMPOWER trial of cemiplimab, an anti–PD-1 agent, in patients with recurrent, persistent, or metastatic cervical cancer who had disease progression with prior platinum-based chemotherapy have for the first time showed that anti–PD-(L)1 therapy can produce better outcomes than chemotherapy in this setting (39). This trial evaluated cemiplimab monotherapy vs investigator choice of single-agent chemotherapy; all patients had received one prior line of therapy, and 40.8% had received more than one prior therapy. In the overall patient population, an improvement in median OS from 8.5 months with chemotherapy to 12.0 months with cemiplimab (*P*<0.001) was reported, and the trial was stopped early (39, 96, 113). Median PFS was 2.8 months with cemiplimab and 2.9 months with chemotherapy (hazard ratio, 0.75; 95% CI, 0.63-0.89; *P*=0.00048); ORRs were 16.4% and 6.3%, respectively. Treatment-related AEs occurred in 56.7% of patients receiving cemiplimab, including 14.7% with grade 3/4 events; the most common treatment-emergent AEs were anemia (25.0%), nausea (18.3%), and fatigue (16.7%) (39). However, this study did not select patients by PD-L1 status, and therefore assessment by PD-L1 subgroup is not available.

In the EMPOWER trial, cemiplimab resulted in a higher ORR in patients with squamous cell carcinoma than in those with adenocarcinoma (17.6% vs 12.3%). These results support a general trend that response rates in patients with adenocarcinoma are typically lower than those in patients with squamous cell carcinoma (3, 39, 114). This has been particularly evident with anti–PD-(L)1 therapies in the second- or later-line setting. In the NRG-GY002 trial, nivolumab treatment resulted in one partial response in 15 patients (6.7%) with squamous cell carcinoma, but no responses occurred in the six patients with adenocarcinoma (115). In a phase 2 study with balstilimab monotherapy in patients with at least one prior chemotherapy, responses were seen in 15 of 85 patients (17.6%) with squamous cell carcinoma and in six of 48 (12.5%) with adenocarcinoma (93). In the KEYNOTE-158 trial, only one of five patients with adenocarcinoma had a response, making it difficult to draw any conclusions; 11 of 92 patients (12%) with squamous cell carcinoma had a response (37). Median OS was improved with cemiplimab in both histological types compared with single-agent chemotherapy (39, 113). The improvement in median OS benefit was numerically higher in adenocarcinoma than squamous cell carcinoma when compared with respective chemotherapy results (6.3 vs 2.3 months). Further analysis of these treatments in these histologic subtypes is needed.

The results to date demonstrate that while anti–PD-(L)1 therapies have efficacy as second- or later-line therapy for cervical cancer, not all patients benefit from therapy. Furthermore, resistance to anti–PD-(L)1 therapy remains a concern. Therefore, therapies that address mechanisms of resistance are needed to further improve outcomes in more patients.

#### Combination Therapies

Studies of combinations of anti–PD-(L)1 agents with other checkpoint inhibitors (inducible T-cell costimulator, cytotoxic T-lymphocyte protein 4 [CTLA-4], T-cell immunoreceptor with immunoglobulin and immunoreceptor tyrosine-based inhibition motif domains), VEGF inhibitors (bevacizumab, cabozantinib, apatinib), and HPV vaccines (ISA101, GX-188E) reflect the importance of targeting multiple pathways to improve treatment outcomes (**Tables 1** and **2**). While clinical trials of many of these combinations are ongoing, the most robust evidence exists for combinations of anti–PD-(L)1 and anti–CTLA-4 agents. Different combinations of anti–PD-(L)1 and anti–CTLA-4 agents have shown ORRs between 20.6% and 36.4% in patients who had received prior systemic therapy (**Table 1**). However, limited efficacy in specific histological types of cervical cancer emphasizes the challenges observed previously with anti–PD-(L)1 monotherapies. Similar to balstilimab monotherapy (93), the combination of balstilimab and zalifrelimab showed lower response rates in patients with cervical adenocarcinoma than those with squamous cell carcinoma (8.8% vs 32.6%) (102).

Two small phase 2 studies have evaluated combinations of anti–PD-(L)1 agents and VEGF inhibitors in patients with at least one prior systemic therapy (101, 103). Due to small sample sizes and the absence of a comparator arm, results of these studies need to be validated in larger trials. Several studies have demonstrated that poly (adenosine diphosphate–ribose) polymerase (PARP) inhibitors have antiangiogenic activity, the ability to promote infiltration of CD8+ T cells, and a role in maintaining genomic stability (116–120). In addition, PARP inhibitors can decrease TGF-β expression, leading to inhibition of EMT and fibrosis (121, 122). Based on these observations, two phase 2 studies are examining combinations of anti–PD-(L)1 agents with PARP inhibitors (niraparib, olaparib) (**Table 2**). One phase 2 study has shown promising activity with simlukafusp alfa, a bifunctional antibody, in combination with anti–PD-L1; however, considerable toxicity seems to be associated with this combination, with 63.4% and 29.8% of patients reporting grade 3 and 4 treatment-related AEs, respectively (106).

#### Dual Targeting Therapy to Inhibit TGF-β and PD-L1

As previously described, in addition to PD-L1, TGF-β may play a significant and related but nonredundant role in the underlying pathophysiology of cervical cancer, providing a rationale for inhibiting both pathways simultaneously. Bintrafusp alfa is a first-in-class bifunctional fusion protein composed of the extracellular domain of the human TGF-β receptor II (TGF-βRII or TGF-β “trap”) fused via a flexible linker to the C-terminus of each heavy chain of an IgG1 antibody blocking PD-L1 (anti–PD-L1) (123–125). Bintrafusp alfa is designed to block TGF-β signaling by “trapping” all TGF-β isoforms (β1, β2, and β3), and this “trap” function is physically linked to PD-L1 blockade in the TME (123, 124). Bintrafusp alfa targets tumors via simultaneous blocking of the immunosuppressive TGF-β and PD-L1 pathways within the TME and is the only agent currently under investigation in cervical cancer that targets both pathways. Studies in murine squamous cell carcinoma models showed that anti–PD-(L)1 therapy rarely led to complete regression, but that adding anti–TGF-β synergistically enhanced antitumor responses. The synergy was partly driven by anti–TGF-β–mediated suppression of anti–PD-(L)1 resistance and by EMT attenuation and immunosurveillance stimulation (109, 126). In mouse models, the combination of bintrafusp alfa and chemotherapy significantly reduced tumor growth relative to either treatment alone (123). In these models, bintrafusp alfa also significantly increased T cells, natural killer cells, macrophages, and dendritic cells and decreased neutrophils and myeloid-derived suppressor cells in tumor tissue compared with anti–PD-L1 or a TGF-β “trap” control (123). Furthermore, as noted above, upregulation of TGF-β signaling and expression in the TME after radiation and chemotherapy treatments may also attenuate the efficacy of, or even promote resistance to, these anticancer therapies (42–44).

To date, bintrafusp alfa is the only agent targeting both TGF-β and PD-L1 to demonstrate efficacy in pretreated recurrent or metastatic cervical cancer. Two clinical studies, INTR@PID 001 (NCT02517398) and NCI 012 (NCT03427411), have evaluated bintrafusp alfa in cohorts of patients with pretreated recurrent or metastatic cervical cancer. In a cohort of 25 heavily pretreated patients with recurrent or metastatic cervical cancer from the phase 1 INTR@PID 001 clinical trial who had disease progression with prior platinum-containing chemotherapy, the ORR was 24.0% per investigator assessment. One patient had a delayed partial response after initial disease progression (127). In a *post hoc* pooled analysis of patients with recurrent or metastatic cervical cancer from the INTR@PID 001 study and a phase 2 single-center study (NCI 012), the ORR was 28.2% (95% CI, 15.0%-44.9%) (94). Responses to bintrafusp alfa were long-lasting, with a median DOR of 11.7 months (range, 1.4-41.2 months), and were observed irrespective of tumor histology or prior bevacizumab treatment. In contrast, anti–PD-(L)1 therapies tend to be less effective in patients with adenocarcinoma (93, 102, 115) and those who have been exposed to bevacizumab (37, 102). Additionally, the safety profile of bintrafusp alfa in patients with heavily pretreated recurrent or metastatic cervical cancer who had disease progression with platinum-containing chemotherapy was consistent with that of anti–PD-(L)1 therapies, except for treatment-related AEs known to be associated with TGF-β inhibitors (e.g., localized skin lesions including keratoacanthomas) (94, 109).

Bintrafusp alfa has also shown potential to treat other rare HPV-related tumors, such as anal, rectal, and vaginal cancer in INTR@PID 001 and NCI 012 trials; the overall ORR was 28.0% in patients with HPV-associated malignancies (109, 128). Anti–PD-1 therapy with nivolumab has also shown a promising response rate of 20.0% in patients with vaginal or vulvar cancers in CheckMate 358 (90).

These data indicate the potential benefit of bintrafusp alfa monotherapy in the postplatinum recurrent or metastatic setting. This is being explored further in a large phase 2 trial (NCT04246489). In addition, a phase 1 study is investigating the safety of bintrafusp alfa in combination with other anticancer therapies in patients with locally advanced or advanced cervical cancer (NCT04551950). Preliminary results from this study showed no new safety signals, and two partial responses among six patients who had a tumor assessment in patients with advanced cervical cancer (87).

## Conclusions

Treatment options are limited in patients with persistent, recurrent, or metastatic cervical cancer. Although several immunotherapies, including anti–PD-(L)1 agents, have shown clinical activity in persistent, recurrent, or metastatic cervical cancer, response rates are relatively low and vary based on PD-L1 expression and tumor histology. Agents that target immunosuppressive mechanisms within the TME associated with HPV infection, such as bintrafusp alfa, which simultaneously targets the TGF-β and PD-L1 pathways, are under investigation. Promising results were observed with bintrafusp alfa in phase 1/2 trials in patients with persistent, recurrent, or metastatic cervical cancer.

## Future Perspectives

Although bintrafusp alfa has shown promising results in patients with persistent, recurrent, or metastatic cervical cancer in phases 1-3, strategies for patient selection, including assessment of PD-L1 status, tumor histology, and TGF-β signature profile, are necessary. In the phase 1 INTR@PID 001 clinical trial, PD-L1 expression was detected by immunohistochemistry in a fraction of patients; a threshold of 1% was used to characterize tumors as PD-L1 positive (≥1%) or negative (<1%) (109). Treatment responses occurred irrespective of PD-L1 status. In a pooled analysis of patients from the INTR@PID 001 and NCI 012 trials, responses to bintrafusp alfa were observed irrespective of tumor histology (94), although a systematic approach in a larger trial might shed additional insights.

The TGF-β signature profile can identify patients with gynecologic cancers who are likely to benefit from immune checkpoint inhibitors (129). This approach may also be used to select patients who may respond to bintrafusp alfa. Another approach can be to investigate if tumor mutational burden (TMB) can be used as a biomarker for this bifunctional agent; TMB has been demonstrated to be a useful biomarker for immune checkpoint inhibitor therapy across cancer types (130). Future trials are needed to clarify the role of biomarkers to identify the patient population that would benefit from bintrafusp alfa treatment.

## Author Contributions

All authors contributed to the conception and design of the manuscript and revised, read, and approved the submitted version.

## Funding

This work was funded by Merck (CrossRef Funder ID: 10.13039/100009945) and was previously part of an alliance between Merck and GlaxoSmithKline.

## Conflict of Interest

KF reports institutional research funding from Merck and MSD. AO reports institutional research funding from AbbVie Deutschland, Abililty Pharmaceuticals, Advaxis, Aeterna Zentaris, Amgen SA, Aprea Therapeutics AB, Bristol Myers Squibb, Clovis Oncology, Eisai, Immunogen, Millennium Pharmaceuticals, MSD de España SA, Pharmamar SA, Regeneron Pharmaceuticals, Roche, and Tesaro; served on advisory boards for AstraZeneca Farmacéutica Spain SA, AstraZeneca KK, Clovis Oncology, Corcept Therapeutics, Deciphera Pharmaceuticals, Eisai, Merck, Roche, GlaxoSmithKline, Got It Consulting SL, Immunogen, KL Logistics, Medison Pharma, MSD de España, Mersana Therapeutics, Novocure GmbH, Pharmamar SA, prIME Oncology, Roche Farma, Shattuck Labs, Sutro Biopharma, Tesaro Bio GmbH, Tesaro Bio Spain SL, and Tesaro; and received travel or accommodation expenses from AstraZeneca, Pharmamar, and Roche. LR received honoraria from BluePrint Oncology, CurioScience, Physicians’ Education Research, and Products in Knowledge; reports consulting or advisory role for Agenus, AstraZeneca, Clovis Oncology, Merck, Genentech/Roche, GOG Foundation, MSD, Mersana, Myriad Genetics, Novartis, Rubius Therapeutics, and Seagen; participated in speakers bureau for AstraZeneca, MSD, and Tesaro; and reports institutional research funding from Aivita Biomedical, Akeso Biopharma, AstraZeneca, GEICO, Genentech/Roche, MSD, On Target Laboratories, Pfizer, and Tesaro. LO reports employment, at the time of the study, with EMD Serono Research & Development Institute, Inc, Billerica, MA, USA, an affiliate of Merck KGaA. CV reports employment with Merck.

The remaining authors declare that the research was conducted in the absence of any commercial or financial relationships that could be construed as a potential conflict of interest.

The authors declare that this work was funded by Merck (CrossRef Funder ID: 10.13039/100009945) and was previously part of an alliance between Merck and GlaxoSmithKline. The funder had the following involvement with the study: two authors (L.S. Ojalvo and C. Valencia) were employees of EMD Serono Research & Development Institute, Inc, Billerica, MA, USA, an affiliate of Merck KGaA.

## Publisher’s Note

All claims expressed in this article are solely those of the authors and do not necessarily represent those of their affiliated organizations, or those of the publisher, the editors and the reviewers. Any product that may be evaluated in this article, or claim that may be made by its manufacturer, is not guaranteed or endorsed by the publisher.
